# Endogenesis and empowerment: logic, practical mechanism and sustainable path of rural minor pension institutions —based on the qualitative study of four villages in the Midwest of China

**DOI:** 10.3389/fpubh.2026.1779131

**Published:** 2026-03-31

**Authors:** Peng Fan, Ruobing Fa, Changqing Wang

**Affiliations:** School of Medical Administration, Nanjing Medical University, Nanjing, China

**Keywords:** endogenous development, geochemical, old-age in rural areas, practical logic, qualitative study, small and micro-institutions for the older adults, sustainability

## Abstract

**Introduction:**

This study explores the formation logic, operational mechanisms, and sustainable pathways of rural micro-pension institutions in central and western China.

**Methods:**

Using qualitative research methods, four typical villages were selected. Data were collected through 46 in-depth interviews and participatory observation, and analyzed using a constructivist grounded theory approach.

**Results:**

The results showed that this model operates through a triple mechanism of “low-capital embedding–emotionalized services–community-based collaborative governance,” forming a low-cost, highly embedded “quasi-public” service network. This network effectively fills the vacuum left by the formal older adults care system in rural grassroots communities. However, it also faces structural challenges, including a lack of legitimacy, insufficient professionalism, and weak risk resilience.

**Discussion:**

Based on these findings, a five-in-one empowerment framework of “conceptual remodeling–institutional unbinding–service support–risk sharing–industrial integration” is proposed. This study provides a grassroots innovation model and policy insights for older adults care in resource-constrained environments. However, the external validity of the findings requires cautious generalization due to limitations in case numbers and the qualitative methodology.

## 1 Introduction

China is experiencing the largest and fastest population aging process in the world, characterized by a distinct “urban–rural inversion” phenomenon. According to data from the seventh national census, 120 million people aged 60 and above live in rural areas, accounting for 23.81% of the total rural population (1). The proportions of older adults aged 60 and above and those aged 65 and above in rural areas are 7.99 and 6.61 percentage points higher than in urban areas, respectively (20, 25). It is projected that by 2028, the proportion of older adults in rural China will exceed 30%, 11 percentage points higher than in cities (2).

Against the backdrop of urbanization and population mobility, intergenerational separation in rural households has intensified, leading to a sharp decline in the traditional family-based older adults care function and resulting in a large number of “left-behind older adults” and “empty-nest older adults” groups. Concurrently, the rural older adults care service supply system faces significant shortcomings (3). Public nursing homes have limited coverage, market-oriented older adults care institutions are difficult to popularize due to high costs and detachment from rural communities, and various mutual support models for older adults are often unsustainable due to funding and institutional issues. The issue of rural older adults care has thus evolved from a family risk to a major social livelihood and governance challenge.

In this context, a type of small-scale, informal, and locally rooted micro-older adults care institution has quietly emerged and spread in rural areas of central and western China (4). These institutions are mostly run by villagers who use their idle houses, typically with fewer than 10 beds, serving disabled, semi-disabled, or empty-nest older adults in their own or neighboring villages. Due to their extremely low cost, high flexibility, and deep local affinity, they have become a pragmatic choice for many rural families to address the older adults’ care crisis, providing a vivid example for observing how grassroots society in China independently responds to such challenges.

The Third Plenary Session of the 20th Central Committee of the Party proposed actively responding to population aging, improving the development of the older adults’ care industry and its policy mechanisms, and accelerating efforts to address shortcomings in rural older adults care services (5), highlighting policy-level attention to this issue.

Current academic discussions on rural older adults care primarily revolve around the dual axes of the state and the market, focusing on macro-policy design or standardized service models. However, there is a significant lack of attention to the spontaneously emerging, small-scale, and informal older adults care practices in rural areas, particularly in terms of in-depth analysis of their endogenous motivations, operational logic, and conditions for sustained existence. By reviewing literature from the past 5 years and key foundational studies, three main research pathways and practical models can be summarized.

First, the resource input pathway emphasizes the fiscal responsibility of the government, advocating increased investment in public facilities or subsidies to address rural older adults care needs (6). While this approach focuses on basic guarantees, it often overlooks the mobilization potential and efficiency of existing grassroots resources and is easily constrained by local fiscal limitations.

Second, the technology empowerment pathway promotes the use of digital technologies (such as smart older adults care and blockchain) to enhance service efficiency and transparency in management (7). However, this pathway typically assumes a certain level of infrastructure and digital literacy, which may not align with the realities of many rural areas in central and western China and could exacerbate new inequalities.

Third, the institutional optimization pathway focuses on top-level institutional design, aiming to regulate the older adults’ care market by improving legal and policy frameworks (8). While its long-term value is undeniable, an overemphasis on formalization may inhibit the vitality of grassroots adaptive innovations.

In parallel, domestic practices have formed three typical models: public welfare-oriented institutions (e.g., township nursing homes), which primarily serve special-needs groups such as Five-Guarantee Households but have limited coverage and service scope (9); market-operated older adults care institutions, which, despite providing basic care services, face low acceptance due to high costs and poor community integration (10); and village community mutual support models, which often rely on civil affairs system day-care projects but frequently remain at a conceptual level due to insufficient funding, misalignment with needs, and a lack of sustainable incentives (11).

Recent interdisciplinary research has further enriched our understanding of relevant dimensions. Studies on the mental health of left-behind older adults in China highlight the critical role of emotional companionship and community integration, aspects often neglected by resource- or technology-driven models (12). Research on sustainable development reveals the inherent tension between pursuing formal efficiency and preserving the social capital embedded in informal community networks (13). Studies on trauma-informed care remind us to pay attention to the principles of safety, trust, and empowerment in caregiving environments (14). Analyses of corporate risk management (15) indirectly illuminate the governance and financial vulnerabilities that informal institutions may face. Furthermore, foundational sociological theories on embeddedness (16) and network governance (17) provide important lenses for understanding how informal institutions operate within and are shaped by local social structures.

In summary, although existing research offers diverse perspectives, significant gaps remain. First, there is a lack of mechanism-based explanations grounded in in-depth field investigations that clarify how rural micro-older adults care institutions navigate specific policy and cultural environments within the state-market-society triadic interaction field and achieve a dynamic balance between structural constraints and individual agency. Second, a systematic analytical framework has yet to be established to identify the core challenges such models face in the absence of formal recognition (e.g., legitimacy dilemmas, gaps in professional capabilities, weak risk resilience) and explore which empowerment pathways can promote their standardization and sustainable development.

Therefore, the overall objective of this study is to systematically explore the formation logic, operational mechanisms, and sustainable pathways of rural micro-older adults care institutions in central and western China. Specifically, it aims to address the following core research questions:

How do structural conditions and subjective agency interact within such institutions under specific policy and cultural environments? What is the source of their endogenous motivation?

How does this model operate concretely? What are the core mechanisms that enable low-cost operation, build community trust, and meet the needs of older adults?

What key constraints and risks does this model face in the absence of formal institutional recognition and professional support? Through what empowerment pathways can its standardization and sustainable development be achieved?

To address these gaps, this study situates rural micro-older adults care institutions within the analytical framework of the state-market-society triadic interaction. Through qualitative investigation in four villages in central and western China, it delves into their endogenous logic, practical mechanisms, and empowerment pathways, aiming to theoretically expand the understanding of informal, community-based older adults care models and provide evidence-based references for policy-making.

This study not only aims to comprehensively present the landscape of this grassroots older adults care practice but also hopes to provide theoretical insights and practical references for understanding the resilience of Chinese rural society, the vitality of grassroots innovation, and the construction of an inclusive older adults care service system through mechanism analysis and typological comparison. It further seeks to contribute wisdom for categorized governance to the development of a multi-level, diversified, and resilient older adults care service system in rural China.

The structure of this study is organized as follows: the second section (Research Methods) details the research design and implementation process. The third section (Research Findings) systematically presents the practical logic, core mechanisms, and typological characteristics of rural micro-older adults care institutions. The fourth section (Discussion) engages in an in-depth dialogue between the research findings and existing theories. The fifth section (Conclusions and Implications) summarizes the research findings, proposes policy recommendations, and outlines the limitations and future directions of the study.

## 2 Materials and methods

Understanding the social phenomenon of rural micro-older adults institutions embedded in local situations must be based on deep immersion in their practice field and a careful grasp of the world of meaning of the actor. Therefore, the methodology adopted qualitative research as the core and case comparison as a strategy. This study employs an embedded multiple-case comparative research design, selecting typical cases based on the principle of maximum variation to enhance the external validity and theoretical saturation of the findings. The research was conducted intensively in August 2024 and has been approved by the Ethics Committee of Nanjing Medical University (Approval No. (2022) 960). All participants signed consent forms, interview content was anonymized, and data were handled with strict confidentiality.

### 2.1 Study design

This study used purposive sampling to select four villages in central and western China. The selection criteria are as follows: First, the villages must have typical practices of small-scale older adults care institutions; Second, they should cover different levels of economic development (including impoverished and average villages); Third, they should reflect different governance models (such as village collective-led, individually managed, and cooperative forms). The principle of maximum variation was adopted to enhance the richness of the findings and theoretical saturation. Although the sample size is limited, informational saturation was achieved through in-depth interviews and participatory observation. As a multiple-case comparative study, the comparison dimensions included resource endowment, actor composition, governance structure, and service models. Cross-case comparison was used to identify common mechanisms and typological differences (see Table 1).

**Table 1:** Basic information and case selection logic of field investigation points.

Field point	Areas to which they belong	Proportion of the population over 60 years of age	Economic form	Typical mode	Rationale for selection
Village H	Central Plains (Henan)	28.5%	Traditional agriculture dominates; some go out to work	Village doctor-led combination of medicine and cultivation	To investigate how professional resources (village doctors) embed and guide small and micro-older adults people.
Village X	Northwest China (Shaanxi)	26.8%	Characteristic planting, rural tourism	Mutual support for older adults in cooperative construction	To investigate how collective organization and economic cooperation support the sustainability of old-age services.
Village Y	Southwest China (Sichuan)	30.2%	Left-behind phenomenon is prominent in large villages exporting labor services	Linkage type of family care point	This study investigates how the decentralized network is constructed in areas with severe population hollowing.
Town C	Northwest China (Gansu)	32.1%	Traditional handicrafts and the new business state of electronic commerce	Local fusion type of “taking work as substitute”	This study examines how traditional cultural resources and modern industry can empower older adults.

### 2.2 Data collection method and process

Data were collected intensively in August 2024, using the “triangular mutual syndrome” strategy combined with various methods. The first is semi-structured in-depth interviews. An interview outline was designed around the study questions but remained open, and 46 valid interviews were completed. The interview objects were classified, and their core concerns were as follows: institutional operators (8 persons). The main focus of the interviews was entrepreneurship motivation, operating costs, income structure, relationship with family/village committees, difficulties, and strategies faced. Nurses (12 persons) were interviewed primarily about practice, job content, skill acquisition, emotional interactions with older adults, and work-related cognition. Admission of the older adults (14 people), primarily interviewed about the admission decision-making process, life experience, evaluation of services, and links with native families and communities. Older adults (6 persons) were primarily interviewed about reasons for choosing this model, cost burden, perceptions of services and safety, and interactions with operators. Village and township cadres (6 persons) were primarily interviewed on their views on the model, policy implementation, regulatory roles, support provided, or regulatory pressures faced. All interviews were recorded with consent and transcribed verbatim into text to form a core database. First, the research team employed a back-translation process to verify the accuracy of key concepts and semantic meanings when translating interview quotations from Chinese to English. Second, participatory observation. The study team members were assigned to four field sites, each stationed in the village for 5 days, to eat and participate in activities with the institutional older adults, observe the daily care process and nurse-older adults interactions, family visit scenarios, village contacts, etc. Detailed observation logs were recorded to capture nonverbal information and contextual details. Third, document and physical data collection. This study collected simple account books, admission agreements, relevant clauses of village rules, residents’ agreements, guidance opinions issued by local governments, propaganda materials, and so on.

### 2.3 Data analysis and coding strategy

Data collection and analysis were carried out synchronously. Systematized coding was conducted with the help of NVivo 12 software, using the constructivist grounded theory and subject analysis of primary coding (open coding). The hanging theory presupposes that all interview transcripts and observation logs are read and tagged line by line to form nearly 400 initial codes. For example, use their old houses, do not pay rent, are villagers, and know the bottom line. The old man is homesick, so his son takes him back to live for 2 days. The fire department said we are not qualified, but did not really shut down. The town doctor comes to measure blood pressure once a month. Secondary coding (spindle coding). The initial code was repeatedly compared, and the association was found, which was grouped into 18 more abstract genera. For example, the code of using one’s own old house, relying on family, and consuming the food grown by oneself is classified as endogenous acquisition of resources and low-cost conversion. The code of knowing the root, seeking the village head for advice, and being unable to hide away is classified as a trust-and-supervision mechanism based on an acquaintance society. Codes such as fire inspection failure, not licensed, and fear of being unable to pay for accidents are classified as lack of legality and risk anxiety. Tertiary coding (selective coding). In all genera, the core genera that can dominate other genera are identified, and the logical relationships between them are sorted out. Finally, we abstracted endogenous practice as the core category, and constructed a dynamic analysis framework around it that includes structural conditions, endogenous resources, practice strategies, practical effectiveness, and dilemmas (see Table 2).

**Table 2:** Example of tertiary coding process (partial).

Original statement (representative citations)	Primary coding (open coding)	Secondary genus (spindle code)	Core category (selective coding)
“It’s my old house, no rent.” “My son works outside, and I take care of two older adults people at home, which is also a bit of income.” “If you cannot finish your own food, give them some, too.”	a1 Use of owned property a2 Part-time family workforce a3 Food self-sufficiency	Endogenous access to resources and low-cost transformation	Endogenous practice
It is all in one village. If he’s not good for the old man, what will he do in the village? “If we have anything to do, we will ask the village mayor for comment.” “His grandfather and my father are cousins.”	b1 Reputation constraints b2 Authoritative village mediation b3 Endorsement of kinship	Trust and supervision mechanism based on an acquaintance society	
“The fire inspection said we were not qualified, but it did not really shut down.” “We have no proof; we are not sure.” “I hear a house in the next county has been banned.”	c1 Regulatory ambiguity c2 Legitimacy Anxiety c3 Policy uncertainty	Lack of legitimacy and risk perception	
“When the old man was homesick, he sent his son back for 2 days.” “There are fewer people and cheaper fees when farmers are busy.” “Some need to take medicine, some do not, we all write it down in the book.”	d1 Elastification of services d2 Seasonal adjustment d3 Personalized Care Record	Flexible requirement response strategy	
“Watching TV together, pulling the family together, is like family members.” “She called me ‘Auntie,’ and I thought she was my aunt.” “We make dumplings together during the holidays.”	e1 Routinization of Emotional Interactions e2 Construction of phylogenetic relationship e3 Festivals are held intensively	Deep Embedding of Emotional Labor	
“Doctors in town take their blood pressure once a month.” “Call 120 if you are sick, but we are far from here, and we are in a hurry.” “We do not know doctors, we are afraid of an accident in the middle of the night.”	f1 Loose link to external health resources f2 Delayed rescue response f3 Medical Ability Anxiety	Vulnerability of medical support	
“In case the old man falls, we cannot afford it.” “There was an old man who left last year, and his family did not bother, but we did give him some thought.” “I want to get insurance, but I do not know which one can cover us.”	g1 Unexpected Compensation Pressure g2 Informal compensation mechanisms g3 Missing insurance products	Operational risk and financial fragility	

To enhance the analytical credibility, the coding process was independently conducted by two researchers, with disagreements resolved through team discussions. Triangulation was also employed to cross-verify data from interviews, observations, and documents. After completing the topic coding, each case was subjected to an in-depth “process-event” analysis to delineate a panoramic view of its emergence to operation. Then, a systematic cross-case comparison was conducted to identify commonalities and characteristics of mechanisms across models, and to test and enrich the theoretical framework in specific situations.

## 3 Results

### 3.1 Practical logic and multidimensional mechanism of rural miniature pension institutions

Based on the deep analysis of field data, this study reveals that the practice of rural micro-pension institutions is a complex adaptive system embedded in the texture of local society. The core logic is through the creative transformation of local resources and the ingenious application of local rules, in the gap between the shortage of formal system supply, open up a low-cost, high-endowment service supply path.

The rise of the small and micro-institutional pension model is not accidental. It is inseparable from the government’s policy support, the adaptation of market demand, and the intensification of rural empty nesting. It is the result of the intersection of the rigid pressure on the demand side and the supply side factor conditions under the specific policy and cultural environment, embodying a “pressure-response” type of adaptive innovation. In the demand-side interview, high-frequency expressions such as “the children are out, a person falls at home, and no one knows” (village Y, Mrs. Wang, 78) and “go to the county nursing home, it takes two or three thousand a month, our pension is only more than 100, cannot afford to live at all” (village X, Mr. Li, 72) and so on. This clearly reveals the double extrusion of the empty nesting of family pension function and market service economy, which constitutes the fundamental motive force for the new model. On the supply side, a large number of idle houses and farmhouses in the countryside, and the labor force of middle-aged and older women is idle due to the inability to care for their families. The residual but effective mutual assistance ethics in the village provide a material and social basis for the birth of small and micro-aged institutions. Regarding policy and cultural environment, the policy orientation of national encouragement to invigorate idle assets in rural areas to develop old-age services (21), together with the current situation in which local finance is unable to build large-scale old-age facilities, constitutes a vague space for coexistence of “encouragement innovation” and “incapable of organizing.” However, the old people’s inclination to “leave home and not leave the village” provides cultural legitimacy for this mode of “old age at home.” The grass-roots society in China is local. Against the background of peasants as living people who gather in villages and settle down for generations, forming a local society born in Si and dying in Si (18), older adults are more willing to retire in such an “acquaintance society.”

Specifically, in the Midwest, the rise of small and micro-institutions for older adults is not accidental. On the demand side, the problem of aging and left-behind is serious, and the economic base is weak, which forces innovation of low-cost old-age. Per capita income in the rural areas of the central and western regions is lower than in the east, and the cost of old-age care in traditional institutions exceeds the affordability of most families. Small and micro-older-age pension institutions use their own houses, land, and labor, significantly reducing costs and becoming a pragmatic choice under economic pressure. The problems of labor outflow and the left-behind older adults are prominent. In recent years, young and middle-aged people have gone out to work in large numbers in the central and western rural areas, and the proportion of older adults left behind is high, which has seriously weakened the function of the family pension. By integrating the surplus labor force in the village and constructing a mutual assistance network, small and micro-institutions can alleviate the “hollow” dilemma. On the supply side, land, housing, labor, and other factors are sufficient, and the space for transformation is large. Homestead policy is relatively loose in rural areas of central and western China, and family courtyards generally occupy a large area, which facilitates the transformation into old-age space. A large number of farmhouses remain idle for a long time due to population outflow, and small and small institutions can not only avoid waste of resources, but also create income for the village collectively by transforming vacant houses. The distribution of villages in the mountainous areas of the Midwest is dispersed, making it difficult to cover the centralized old-age institutions. The old-age in small and micro-institutions is distributed within family units, which is more suitable for geographic conditions. Furthermore, rural medical facilities in the Midwest are weak, and professional nursing is difficult to popularize. Small and micro-institutions pay more attention to basic life care and chronic disease management, and better match with local resources. At the policy end, the policy of rural revitalization and rural reform provides the institutional dividend. The central and western regions are the key areas for rural revitalization, and the policy encourages the activation of idle rural resources. For example, in 2019, the Ministry of Agriculture and Rural Areas issued the Notice on Actively and Steadily Carrying out the Vacation and Utilization of Idle Housing and Idle Housing in Rural Areas (19), which uses agricultural houses to develop the old-age industry. After the transfer, the house is transformed into a community for the older population or a well-being center, which achieves the triple-win effect of “farmers’ income increase, collective gain, and old-age quality improvement.” The central and western local governments are under great financial pressure and tend to popularize the “light assets” pension model. For example, the “courtyard retirement and mutual service,” which is popularized in Gansu Province, is subsidized by the government, rather than directly invested in the construction of nursing homes, which not only enliven resources, but also provides general and accessible old-age services, while preserving the old people’s original lifestyle and social circle, with remarkable effects. On the cultural side, traditional concepts and mutual aid networks reduce resistance to implementation. The traditional culture in the central and western rural areas is relatively intact, and older adults are generally excluded from leaving their homeland to stay in urban retirement institutions, and are more dependent on family and neighborhood relationships. The mode of “never leaving home, not leaving the village” in small and micro-institutions fits their psychological needs. The patriarchal network in the middle and western rural areas is relatively tight. The tradition of neighborhood mutual assistance provides the social basis for the mechanisms of “time bank” and “mutual assistance group,” and reduces the organizational costs of old-age services.

### 3.2 Core practice mechanism: a trinity analytical framework

In the process of operation, a set of local, flexible, pragmatic and resilient unique practice mechanism has been formed to ensure its survival and development. This mechanism can be systematically summarized into the following three mutually supportive dimensions.

First, the mechanism of resource activation and cost control. This is the material basis and survival bottom line for the organization to realize economic sustainability. In terms of space resources, operators make full use of policies and local resources to convert their own houses and houses into zero-rent premises, thus avoiding the largest fixed cost expenditure. The start-up funds were highly focused on functional transformation, and the investigation showed that the cost of single-bed aging modification was flexibly controlled at 3,000 to 10,000 yuan. The organization of the labor force shows distinctive characteristics of “internalization” and “low monetization.” The main body of the operation is mostly housewives or middle-aged couples who return home, and labor input is often integrated into family livelihoods rather than into completely market-oriented employment; there were also middle-aged and old-aged women in this village. The monthly salary was generally 1,500–2,500 yuan, significantly lower than the urban market rate, and the employment relationship was deeply embedded in the human network and community reciprocity tradition. In the operation link, the means of subsistence are obtained geographically; the food is partly derived from the vegetable plot and partly purchased from the villagers, which not only reduces the daily expenses but also establishes the economic symbiotic link with the community. An analysis of the accounts of eight institutions showed that their total monthly operating costs ranged from 2000 to 5,000 yuan (depending on scale), and the cost structure was stable. Human cost accounts for 50–65%, and food, water, and electricity account for 20–30%. In terms of fees, 500–800 yuan per person per month, as long as the occupancy rate is stable at 70–90%, 5–15% of small profits can be achieved. This set of cost-control logic enables the organization to remain operational under low charges.

Second, trust production and relationship governance mechanisms. This constitutes the key soft power for institutions to embed in local society and gain informal legitimacy. First, the trust spillover effect is highly dependent on the society of acquaintances. As a community insider, the operator’s personal reputation, family history, and moral image become the most important “social credit collateral.” “All live in a village. If he treats older adults badly, how can he raise his head in front of his villagers?” (Interview with village cadres in Village H). This kind of trust, based on geo-consanguinity, greatly reduces the risk of decision-making for family members and the cost of credit establishment for institutions. Second, informal contracts and flexible services are generally practiced. Written admission agreements are extremely abbreviated, with extensive service details and special arrangements dependent on verbal agreements and tacit understanding. The service content is highly personalized, such as respecting and allowing older adults to continue farming habits, freedom to come and go, and family members visit at any time. This elasticity and precision fit the characteristics of non-standardization of rural pension needs and strong emotional dependence. Third, there exists “fuzzy governance” of village-level organizations. Village committees are usually informed and tacitly, or even implicitly supportive, playing the role of “peacemaker” in the event of minor friction, but rarely formal administrative filing or rigid supervision. This kind of “visible but loosely managed” governance posture provides a survival gap for small and micro-institutions, subject to both endorsement and over-intervention.

Finally, the service supply-and-demand response mechanism. This determines the functional effectiveness and community identity of the institution. In terms of service orientation, the institution is based on the core functions of life care and emotional companionship, rather than professional medical care beyond its own capabilities. Medical needs should be met by establishing loose cooperation with township hospitals, such as relying on their regular visits, and opening emergency green channels. In the service mode, it shows a high degree of flexibility and seasonality, providing “mixed” service packages such as full-care, day-care, temporary hosting, etc.; in the peak busy season, it even actively adjusts the service price or mode to respond to the periodic time and financial constraints of rural families. Especially, emotional labor is deeply embedded in the service process. Nurses and older adults, beyond the simple service contract, also bear the kind of kinship-like neighborhood warmth. Day-to-day interactions such as sunbathing, talking, and recalling the past together constitute an indispensable emotional value in care, which is the humanistic kernel that is difficult to replicate in large-scale, standardized old-age institutions.

Small and micro-pension institutions combine cost control, trust capital, and flexible service, which are deeply rooted in rural socio-economic and cultural soil, forming an adaptive system to make full use of local resources, social networks, and local knowledge. Although the system has limitations in specialty and risk management, at the present stage, it effectively responds to the urgent needs of basic old-age in rural areas at an extremely low organizational cost and a high degree of community fit.

### 3.3 Common features and typical differences of patterns

The practice of rural micro-pension institutions shows distinct regional characteristics and resource dependence. In the deep structure of its operating logic, it shows some common characteristics with universal explanatory power. As the theoretical kernel of a specific old-age model, these common components enable it to transcend specific geographical limits and demonstrate similar vitality and adaptability in different rural social environments. At the same time, based on structural differences in resource endowment, subject composition, and social network, different cases have developed their own unique practice forms and formed identifiable types of patterns. The systematic analysis of these similarities and differences will help more precisely grasp the internal mechanism and possible development trajectory of its operation.

#### 3.3.1 Analysis of common features: bottom logic beyond geography

The practice of micro-pension institutions at different field sites shares the following core commonalities, which constitute the intrinsic stipulation that distinguishes them from formal institutions.

Decapitalization and Localization of Resource Portfolio. All institutions show a very low dependence on market-based factors of production and a high degree of dependence on local non-marketization or semi-market resource mix. This method of resource acquisition follows the principle of minimum cost by reconfiguring and reusing existing resources, thereby transforming fixed costs into sunk costs. For example, village doctors in village H capitalize on personal skills and social prestige; village X functions collectively as an idle asset; village Y combines family surplus labor with space; and town C activates the intangible cultural heritage of older adults. This resource portfolio enables survival in the absence of large-scale external investment and aligns the price of its services with rural older adults’ ability to pay.

The informality and relationship embedding of governance structure. Primary does not rely on hierarchical rules and regulations or formal contracts, but is embedded in a complex network of local social relations, such as day-to-day management, service delivery, and dispute mediation. Relational governance has become the dominant model, trusting local knowledge based on long-term living together, supervising the pressure of moral public opinion dependent on an acquaintance-based society, and coordinating through the informal authority of village-level organizations or community elites. Although this governance method blurs the boundaries between rights and responsibilities, its transaction and execution costs are extremely low, and it is highly adaptable to the situation.

The service content is life-standard and emotion-intensive. The focus of service provision is explicitly on basic life care and emotional companionship, rather than specialized medical care. This orientation is not only a passive choice of limited ability, but also an active response to the core needs of older adults in rural areas. In the interview, someone cooks a hot meal, and someone speaks and looks at familiar people and places, which are repeatedly mentioned, even more important than expected for professional care. Therefore, the process of service contains a great deal of emotional labor; nurses are not only service providers, but also listeners, companions, and maintainers of phylogenetic relationships. This emotional level of depth of satisfaction is the standard; a commercial organization finds it hard to replicate the core value.

Involution and Community Sharing of Risk Response. In the face of a range of operational risks, institutions generally lack formal risk management tools and transfer mechanisms. The risk coping strategy of Primary is characterized by internalization: Primary relies on the operator’s individual double caution, narrows the scope of service to avoid high-risk activities, and, in case of an accident, relies on personal savings, family support, and even local human conditions for internal digestion. Simultaneously, the risks are in fact partially transferred and shared across the community network: family members reduce the cost of prior supervision based on trust, but also bear the possible service defects afterwards; village-level organizations play a key role in dispute mediation and, in fact, bear part of the cost of social stability.

#### 3.3.2 Typing differences: resource endowment and shaping dominant logic

Although the underlying logic is shared, specific practice patterns are differentiated by core resources and drivers. Table 3 systematically compares four typical patterns across five dimensions, Table 4 revealing the mechanism by which typological differences are formed.

**Table 3:** Representative quotations and corresponding mechanisms across cases.

Case	Model type	Core mechanism keywords	Representative quotation/observation summary	Corresponding tertiary coding
Village H (Henan)	Village Doctor-Led Type	Professional trust, Low-cost embedding	“It’s my old house, no rent.” / “Town doctors take blood pressure once a month.”	Endogenous access to resources: vulnerability of medical support
Village X (Shaanxi)	Cooperative Mutual-Aid Type	Collective economy, Collective decision-making	“We use the cooperative’s idle building.” /“During busy farming seasons, some older adults go home to help.”	Endogenous access to resources: seasonal service adjustment
Village Y (Sichuan)	Family linkage type	Social networks, distributed governance	“I take care of two older adults at home, which also brings some income.” / “We all live in the same village; he cannot run away.”	Relationship embedding: trust mechanism
Town C (Gansu)	Work-as-Substitute Type	Cultural empowerment, market linkage	“Older adults make handicrafts, and the e-commerce center helps sell them.” / “We make dumplings together during the holidays.”	Emotional labor; cultural capital activation

This table illustrates the derivation process from raw data to core mechanisms, enhancing analytical transparency and the visibility of the evidence chain.

**Table 4:** Comparison and analysis of four kinds of practical models of small and micro-aged institutions.

Analytical dimension	Village H: village medical leading type	Village X: co-operative type	Village Y: family linkage	Town C: working as a substitute
Core driven logic	Professional authority driving and social integration	Collective action driving and industrial feeding	Market demand driving and entrepreneurial ability	Cultural identity driving and self-realization
	Based on the legal basis of village doctors’ professional skills (medical symbol capital) and personal moral prestige, they have dual motivations to address the problem of community pensions and consolidate their social status.	Relying on cooperatives as a collective economic organization, the old-age service is regarded as a part of collective welfare or community industry to realize the linkage between economic and social benefits.	Initiated by rural energetics (usually middle-aged women) who are keen to identify market demand, replication, and chain-in-house units are essentially a mini-social undertaking.	It is rooted in local handicraft culture, which combines the endowment service with non-material culture inheritance and the remodeling of self-value of older adults to meet the spiritual needs of old-for-do.
Key resource endowments	Human capital and symbol capital	Organizational capital and physical capital	Social capital and space capital	Cultural capital and market capital
	The core comprises the professional abilities of individual village doctors, community reputation, and possible micro-policy subsidies (e.g., Happiness Hospital subsidy).	The core is the collective ownership of idle housing (such as old school buildings), the stable economic income of cooperatives, and possible tourism resources.	The core is to initiate family demonstration effects, management experience, and multiple decentralized family homes mobilized through kinship and geographic networks.	The core is the mastery of handcraftsmanship (non-heritage or local skills) of older adults and the external market channels docked through the township e-commerce center.
Characteristics of governance structure	Parental asylum governance	Governor-style collective governance	Network distributed governance	Project-based participatory governance
	The centralized structure is formed around the individual village doctors; decision-making is highly centralized, and management is family and humanistic.	Collective decision-making shall be made by the cooperative council, implemented specifically by the head of the old-age plate, and income distribution and risk-bearing shall be organized accordingly.	A nuclear family acts as the “headquarters” for brand output and loose coordination; each franchise point operates independently, and village-level organizations assume part of the linkage function.	Township e-commerce center to provide platform and market guidance, older adults form a mutual help group for self-management, and the operation process has a high degree of participation and autonomy.
Key hubs for sustainability	Persistence and publicity maintenance of key personnel	The stability of the main industry profit and the institutionalization of the feeding mechanism	Dynamic balance of standardization, flexibility, and risk joint prevention network	Market competitiveness of cultural products and the sustainability of older adults participation ability
	It is highly dependent on the health, will, and moral consciousness of individual village doctors. If its publicity is weakened to be purely for-profit, the basis of trust may be shaken.	The survival of old-age services depends on the overall profitability of the cooperative and whether the rules of “make-for-profit” can be written into the charter of the organization and observed.	There is a need to find a balance between unifying the service bottom line and respecting the autonomy of each point, and establishing effective internal mutual assistance and risk-sharing mechanisms.	Continuous product innovation is needed to adapt to market changes and to ensure that the hand function and creative enthusiasm of older adults can be maintained to avoid withdrawal due to physical decline.
Core Tension Facing	The Tension of Personalization and Institutionalization	Tension between Economic Benefit and Social Benefit	Scale Effect and Tension of Management Risk	The Tension of Cultural Inheritance and Market Efficiency
	The success of the model depends on one person, and there are obvious risks of “human governance” and an intergenerational inheritance dilemma. How to transform individual authority into sustainable institutional arrangements is a challenge.	When the economic pressure of cooperatives increases, the old-age investment may be squeezed. Independent evaluation and monitoring mechanisms are needed to ensure the implementation of social commitments.	Chain expansion may pose risks of uneven service quality and uncontrolled management. Once a serious accident occurs, it may lead to a crisis of trust throughout the network.	The long production cycle and low efficiency of handicrafts may contradict the pursuit of fast turnaround and low cost in the e-commerce market. Excessive commercialization may erode its cultural connotation and the essence of old age.

#### 3.3.3 Dialectical unity of commonalities and differences: an integrated understanding

By analyzing commonality and difference, we can gain a more integrated understanding of rural micro-pension institutions. On the one hand, common logic is the bottom line of its existence. Regardless of the form changes, the three principles of low-cost embedding, relationship governance, and life-emotional service core constitute the minimum common multiple for small institutions to survive in a resource-scarce rural environment. Any attempt to deviate from these fundamental principles can quickly drive up costs, lose trust, and disengage from demand, making it difficult to sustain. On the other hand, typological differences are the upper limit of their development. Different resource combinations and driving logic determine different development potentials, different bottlenecks faced by various models, and the required focus of empowerment. For example, the problem of institutionalized inheritance is the most urgent problem to be solved; the cooperative type needs to perfect the governance structure to ensure the stability of the old-age investment; the key of the family linkage type is to establish effective quality supervision and a risk-sharing mechanism.; however, the substitute-for-work type needs to find a balance between cultural value and market law. This means that future policy support and empowerment measures must abandon the one-size-fits-all approach and implement fine, differentiated design and intervention. It can be said that the practice of rural micro-pension institutions is a spectrum of diversified innovation unfolding from a unified underlying logic. Understanding its commonalities and differences will not only help evaluate its value and risk more comprehensively, but also construct a multi-level, diversified, and resilient Chinese rural endowment service system, and provide valuable wisdom and classification guidance basis from grass-roots practice.

#### 3.3.4 Pervasive dilemmas and risks faced

Despite their vigorous appearance, small and micro-institutions generally walk on the brink of fragility; their dilemma is structural, manifested in the following four aspects. First, the legal dilemma and regulatory risk. More than 60% of the institutions interviewed lacked any formal permission. They exist in the grey zone of policy and can be shut down at any time due to safety inspections, accident accountability, or policy tightening. Operators generally feel insecure about doing it day-to-day. Second, the low level of specialization and medical risk. Nurses are generally older and less educated, and only about 20% have received short-term basic training (22). The facility has little emergency equipment or medicine. Depending on family members or calling 120 for a sudden illness, remote villages may have a response time of more than 1 h. Weak medical support is the greatest safety concern. Third, financial fragility and operational risk. The low fee model determines that its profit margin is extremely thin. Once older adults fall, get lost, become ill, or experience other accidents, even if the responsibility is not entirely with the institution, based on local conditions and visitation pressure, they often need to pay a large amount of compensation, which may directly lead to the institution’s collapse. Fourth, the intergenerational inheritance dilemma. Operators are mostly middle-aged and older adults people aged 50–65, and most of their children are reluctant to inherit the “hard, responsible, unprofitable” home. The Sustainable Development of Institutions Faces the Threat of Talent Disconnection.

## 4 Discussion

The practice of rural micro-pension institutions offers a distinctive model for addressing aging in rural China—an adaptive program rooted in endogenous resources and community resilience. Our findings showed that this model thrives through a triple mechanism of low-capital embedding, emotionalized services, and community-based collaborative governance, enabling it to operate with minimal external inputs while maintaining deep social embeddedness. This stands in contrast to dominant policy and research paradigms that prioritize formal, resource-intensive, or technologically driven solutions. In what follows, we situate our findings within the existing literature, highlighting points of convergence and divergence, and explain how this study advances understanding of informal, community-based older adults care in resource-constrained settings.

### 4.1 Comparison with existing research paradigms

#### 4.1.1 Contrast with the resource input pathway

Much of the current policy discourse emphasizes increasing government financial investment in rural older adults care, advocating for the expansion of public nursing homes or subsidies for market-based institutions (6, 9). While such approaches are vital for systemic support, they often overlook the capacity and efficiency of grassroots resource mobilization. Our study shows that rural micro-institutions operate with extremely low fixed costs by utilizing idle housing, family labor, and local food sources—resources that are seldom accounted for in formal financing models. This endogenous resource activation represents a form of “asset-based community development” largely absent in top-down, resource-input-oriented literature. Unlike public institutions that face high per-bed construction costs and operational subsidies, micro-institutions achieve affordability through social and spatial embeddedness, a mechanism that formal models struggle to replicate.

#### 4.1.2 Contrast with the technology-driven and system optimization approaches

Recent studies have highlighted the potential of digital technologies (e.g., blockchain, smart older adults care) and systematic institutional design to enhance rural older adults care (7, 8). While these are promising directions, they often assume a certain level of infrastructure, literacy, and institutional capacity that is lacking in many rural areas in central and western regions. Our findings indicate that micro-institutions rely not on advanced technology, but on relational governance and tacit local knowledge—what we term fuzzy governance within acquaintance societies. This aligns with Kaiser’s (10) observations on the endogenous character of rural care, but extends it by detailing how informal contracts, reputation mechanisms, and village-level mediation substitute for formal monitoring and compliance systems. Thus, while technology and system design are important for scaling and risk management, they must be adapted to, rather than imposed upon, existing social structures.

#### 4.1.3 Alignment with community-coordinated and mutual-aid models

Our findings align with emerging research on village-based mutual-aid older adults care (11) and cooperative-driven models (10). Like these studies, we observed that collective action, social networks, and shared cultural norms are critical to sustainability. However, prior work often treats these models as homogeneous or idealizes their community spirit without examining internal tensions and operational fragility. Our typological analysis reveals that even within community-based models, governance structures, resource dependencies, and risk profiles vary significantly. For example, the cooperative model in Village X faces tensions between economic viability and social welfare, while the family-linkage model in Village Y struggles with quality control across decentralized nodes. These nuances are often undertheorized in the literature, which tends to focus on institutional forms rather than processual and relational dynamics.

### 4.2 Explaining divergences: the role of informal legitimacy and adaptive pragmatism

A key divergence between our findings and much of the formal older adults care literature lies in the source of legitimacy and trust. While formal institutions derive legitimacy from state accreditation, professional standards, and legal contracts, micro-institutions derive theirs from embeddedness in local social networks, moral reputation, and community endorsement. This acquaintance-based trust significantly lowers transaction costs and enables operation in a policy grey zone. This reality is often pathologized in regulatory studies as non-compliance but is reframed in our study as adaptive pragmatism.

This perspective aligns with Zhao and Wu’s (18) analysis of grassroots social mobilization in traditional rural settings, but extends it by showing how such mobilization is not merely a cultural residue but a strategic response to institutional voids. The triple mechanism we identify—low-capital embedding, emotionalized services, and community-based governance—functions as an informal institutional substitute that fills gaps left by the state and market. This challenges the dominant “formalization imperative” in older adults care policy and suggests that hybrid governance—blending informal social regulation with light-touch formal support—may be more appropriate in certain rural contexts.

### 4.3 Toward a systematic empowerment framework: aligning challenges with interventions

The structural vulnerabilities of micro-institutions, such as legal ambiguity, medical risk, financial fragility, and intergenerational succession, cannot be resolved through spontaneous community action alone. Based on our diagnostic analysis, we propose a five-dimensional empowerment framework (Table 5) that matches specific observed challenges with targeted interventions, aiming to preserve endogenous strengths while mitigating systemic risks.

**Table 5:** Correspondence between structural challenges and systematic empowerment measures.

Observed challenges	Dimensions of empowerment	Specific countermeasures	Expected outcomes
Emergency response delays	Service support	Establishment of “Village Doctor Contract System” and “One-Click Call Devices”	Reduction of response time to within 30 min
Regulatory ambiguity	Institutional unbinding	Implementation of “Layered Filing System” with clarified baseline standards	Institutions gain legal recognition; regulatory expectations become predictable
Financial fragility	Risk sharing	Introduction of “Comprehensive Liability Insurance for Micro-Institutions” and “Risk Mutual Aid Fund”	Dispersal of compensation pressure from individual incidents
Low professionalization	Service support and talent training	Launch of “neighborhood caregiver” training programs and establishment of health records	Improvement of basic care skills and standardization of health management
Intergenerational succession dilemma	Industrial Integration	Promotion of “Older adults Care, Craftsmanship, and E-commerce” integration to enhance economic appeal	Attraction of young returnees to participate in operations

This framework moves beyond the conventional “resource injection” or “standardization” approaches criticized in the literature (6, 8), and instead embraces context-sensitive, multi-stakeholder, and hybrid governance strategies. For instance, the proposed “layered filing system” offers a pragmatic pathway to legal recognition without imposing prohibitive compliance costs—a middle ground often missing in policy debates.

### 4.4 Theoretical implications: toward an integrated “state–market–society” framework

Our study contributes to the growing scholarly call to move beyond state- or market-centric frameworks and toward an integrated “state–market–society” triad in understanding rural older adults care. While existing literature often pits these sectors against each other (e.g., state failure vs. market failure), our cases show that micro-institutions operate in the interstices of all three, leveraging:

- State tolerance (via tacit village-level support),
- Market logic (through fee-for-service and product sales), and
- Social capital (through kinship and neighborly networks).

This triadic embeddedness enables resilience but also creates vulnerabilities—particularly when one sector withdraws or imposes constraints (e.g., regulatory crackdowns, outmigration, market fluctuations). Future research should therefore adopt a relational and dynamic perspective, examining how these three realms interact in practice rather than in theory.

### 4.5 Policy implications: from standardization to stratified empowerment

Our findings caution against one-size-fits-all policy approaches that seek to standardize or formalize micro-institutions without regard to their ecological diversity. Instead, the empowerment framework outlined in Table 5 supports a shift from compliance-oriented governance to enablement-oriented governance. This perspective remains nascent in the Chinese discourse on rural older adults care policy.

In particular, our emphasis on layered filing systems, community-risk pooling, and cultural-industry integration offers concrete mechanisms for sustaining informality while gradually enhancing safety and professionalism (24). These measures are designed to augment rather than replace the endogenous strengths of micro-institutions, a principle that is often overlooked in top-down pilot projects or technology-driven solutions.

### 4.6 Limitations and avenues for future research

While this study provides a thick description of the logic and mechanisms of rural micro-pension institutions, its qualitative and small-N design limits statistical generalizability. Future research should:

- Employ mixed methods to quantify the prevalence, cost-effectiveness, and outcomes of such models across regions.
- Conduct longitudinal studies to track institutional evolution, survival rates, and the impact of policy interventions.
- Compare cross-national cases of informal older adults care in other Global South settings to identify transferable principles.

Moreover, as digital infrastructure expands into rural China, future studies should examine how smart older adults care technologies can be appropriated and adapted by micro-institutions without undermining their relational core—a tension that remains underexplored in both policy and academia.

## 5 Conclusion

This qualitative study uncovered the endogenous logic and operational mechanisms of rural micro-pension institutions. Key findings are as follows: first, the model achieves low-cost operation and community embeddedness through a triple mechanism of low-capital embedding–emotionalized services–community-based collaborative governance; second, it faces structural challenges such as lack of legitimacy, insufficient professionalism, and weak risk resilience; third, its sustainability cannot rely solely on spontaneous order but requires systematic empowerment. These findings deepen the understanding of informal older adults care practices in resource-constrained environments.

Based on the findings, the following policy recommendations are proposed: First, establish an adaptive regulatory system characterized by ‘categorization, classification, and progressive compliance’ to create legitimate space for micro and small institutions; Second, build a ‘Medicare-nursing integrated, digitally connected’ support network to enhance service capabilities; Third, set up risk compensation funds (23) and insurance mechanisms to strengthen risk resilience; Fourth, promote ‘government-market-society’ collaborative governance to form an enabling system with multi-stakeholder participation. These recommendations provide an actionable framework for local governments and relevant entities.

### 5.1 Limitations and future research

This study has limitations inherent in its qualitative approach, including limited generalizability due to the small number of cases and the lack of longitudinal or quantitative data. Future research should employ mixed methods to validate the findings across broader regions, conduct longitudinal tracking to assess sustainability, and evaluate the real-world effectiveness of empowerment policies and digital support tools for these micro-institutions.

## Data Availability

The original contributions presented in the study are included in the article/supplementary material, further inquiries can be directed to the corresponding author.
